# Chromosome-Level Genome Assembly of *Anthidium xuezhongi* Niu & Zhu, 2020 (Hymenoptera: Apoidea: Megachilidae: Anthidiini)

**DOI:** 10.1093/gbe/evac014

**Published:** 2022-02-12

**Authors:** Dan Zhang, Jianfeng Jin, Zeqing Niu, Feng Zhang, Michael C Orr, Qingsong Zhou, Arong Luo, Chaodong Zhu

**Affiliations:** 1 Key Laboratory of Zoological Systematics and Evolution, Institute of Zoology, Chinese Academy of Sciences, Beijing, P.R. China; 2 College of Biological Sciences, University of Chinese Academy of Sciences, Beijing, P.R. China; 3 Department of Entomology, College of Plant Protection, Nanjing Agricultural University, P.R. China; 4 International College, University of Chinese Academy of Sciences, Beijing, P.R. China; 5 State Key Laboratory of Integrated Pest Management, Institute of Zoology, Chinese Academy of Sciences, Beijing, P.R. China

**Keywords:** bee, genome annotation, comparative genomics, gene family evolution

## Abstract

Anthidiini, a large bee tribe characterized by light-colored maculations, represents nearly 1,000 pollinator species, but no genomes are yet available for this tribe. Here, we report a chromosome-level genome assembly of *Anthidium xuezhongi* collected from the Tibetan Plateau. Using PacBio long reads and Hi-C data, we assembled a genome of 189.14 Mb with 99.94% of the assembly located in 16 chromosomes. Our assembly contains 23 scaffolds, with the scaffold N50 length of 12.53 Mb, and BUSCO completeness of 98.70% (*n* = 1,367). We masked 25.98 Mb (13.74%) of the assembly as repetitive elements, identified 385 noncoding RNAs, and predicted 10,820 protein-coding genes (99.20% BUSCO completeness). Gene family evolution analyses identified 9,251 gene families, of which 31 gene families experienced rapid evolution. Interspecific chromosomal variation among *A. xuezhongi*, *Bombus terrestris*, and *Apis mellifera* showed strong chromosomal syntenic relationships. This high-quality genome assembly is a valuable resource for evolutionary and comparative genomic analyses of bees.

Significance

Many species of Megachilidae are renowned for their ability to pollinate crops and pasture, and for their unusual nesting behaviors. The current lack of high-quality chromosome-level genome resources for the Anthidiini limits our understanding of their evolution and biology. Further, there are few genomic resources for high elevation species. In this study, we present a chromosome-level genome assembly and annotation of *Anthidium xuezhongi*, including also synteny and gene family evolution analysis. Our results represent a valuable resource for further study on the evolutionary biology of Megachilidae.

## Introduction

Among bees, the family Megachilidae, especially the subfamily Megachilinae, contains some of the most important wild pollinators for crops and pasture (Holm 1984; Free 1993; Garibaldi et al. 2013; Richards 2016). The tribe Anthidiini, with yellow or reddish-yellow maculations, is a major group within Megachilinae, with nearly 1,000 species (Michener 2007; Niu et al. 2020; Ascher and Pickering 2021). Anthidiini exhibit intriguing nesting behavior (Michener 2007; Litman et al. 2016). Like many megachilids, they nest in preexisting cavities in various substrates, including both preexistent burrows and man-made cavities, and some species show remarkable plasticity (Hicks 1933, Michener 2007). Some species build their nests on the surface or underside of rocks, stones, or stems (*Anthidiellum*; Pasteels 1977; Gess and Gess 2007). Cells within the nests are also lined with various materials such as resin, soil particles, pieces of leaves, chaff, fibers, and plant trichomes (Banaszak and Romasenko 1998; Michener 2007). Despite being interesting and important bees, no high-quality chromosome-level genomes have been reported from the tribe Anthidiini, and very few chromosomal assemblies are available for bees in general. To date, there are few megachilid genomes, with only three among the _**∼**_70 bee genomes published (accessed on November 2021 from NCBI).

In this study, we report the first chromosome-level genome assembly of Anthidiini, *Anthidium xuezhongi* Niu & Zhu, 2020, collected from Tibet. We annotated the noncoding RNAs (ncRNAs), protein-coding genes, and repeat elements. We performed gene family evolution analyses on *A. xuezhongi* and 13 well-studied insect species from major insect orders. In addition, we analyzed interspecific chromosomal variation relationships among *A. xuezhongi* and the well-studied bees *Bombus terrestris* and *Apis mellifera*. This high-quality genome of *A. xuezhongi* is an important step toward bettering our knowledge of the megachilids, whereas also enabling further comparative studies on nesting biology and behavior.

## Results and Discussion

### Genome Assembly

We sequenced 101.83 Gb clean reads in total, including 31.76 Gb (168×) PacBio reads, 28.74 Gb (152×) Illumina reads, 35.05 Gb (185×) Hi-C reads, and 6.28 Gb transcriptome reads (supplementary table S1, Supplementary Material online). The N50 and mean length of the raw PacBio long reads were 21.29 and 18.58 kb, respectively. Following quality control, 26.40 Gb Illumina reads were retained for the genome survey and subsequent genome polishing. We estimated the genome size with GenomeScope under the 21-k-mer parameter. The genome survey suggests that the sequenced *A. xuezhongi* had a genome size of 188–193 Mb, a single simple peak and statistical analyses showed that its genome had a very low repetitive content and heterozygosity.

**Fig. 1.**
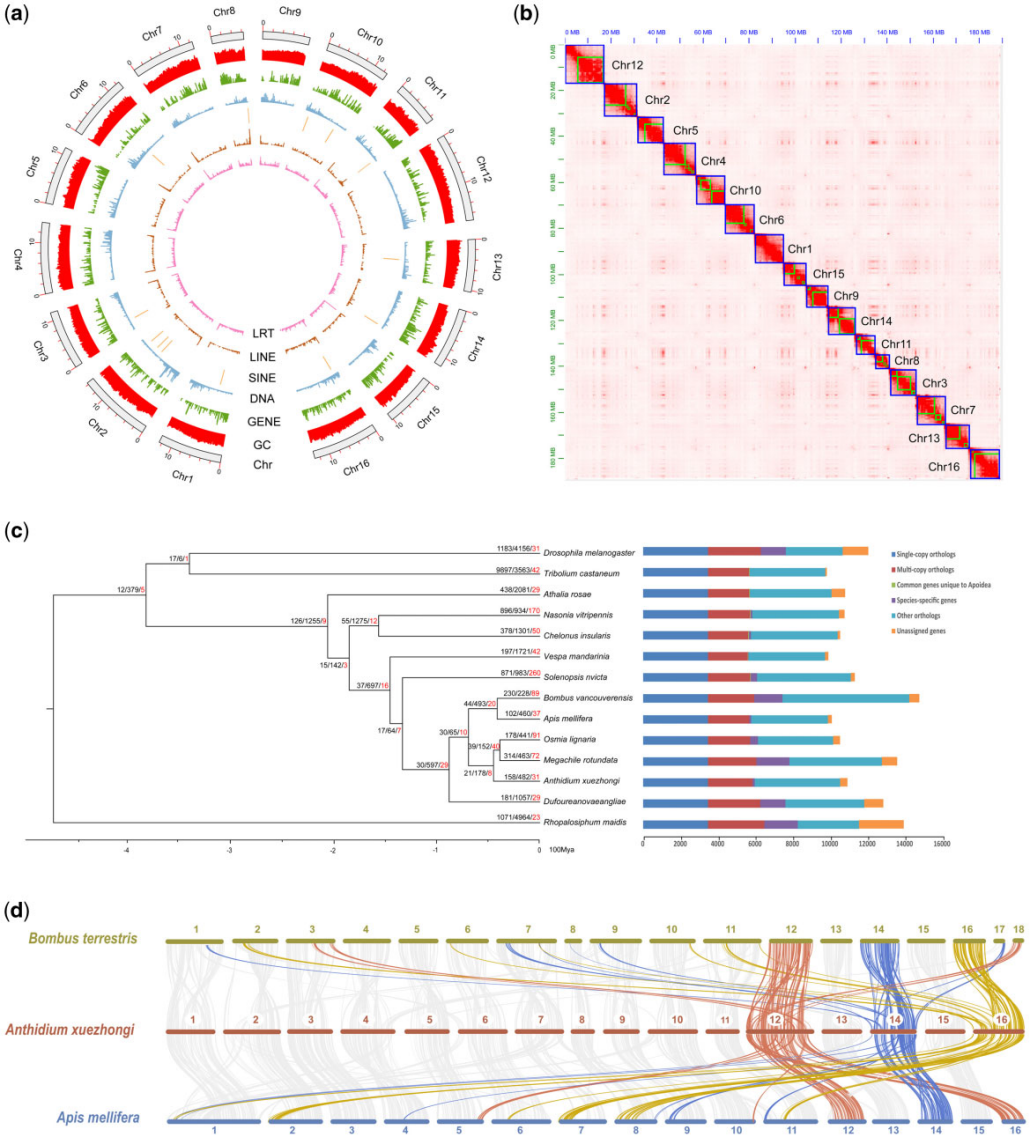
Genome characteristics, gene family evolution, and synteny. (*a*) Circos plot showing the genomic characters of *Anthidium xuezhongi* from outer to inner: chromosome length (Chr), GC content (GC), density of protein-coding genes (GENE), DNA transposons (DNA), SINE/LINE/LTR retrotransposons (SINE, LINE, LRT). (*b*) Genome-wide chromosomal contact map of *A. xuezhongi* with Hi-C data, the blue boxes show super scaffolds and green boxes show contigs. (*c*) Phylogenetic and comparative gene family analyses. Node value represented expansion, contraction, and rapid evolution (red). (*d*) Synteny among *A. xuezhongi*, *Bombus terrestris*, and *Apis mellifera*.

Raw PacBio reads were assembled with Flye, resulting in a 190-Mb assembly with 12.55 Mb scaffold N50 length. After polishing, contaminant removal, redundancy checks, and Hi-C scaffolding, the final *A. xuezhongi* assembly had a length of 189.41 Mb, including 23 scaffolds and 168 contigs, with the scaffold/contig N50 size of 9.28/6.53 Mb and a GC content of 38.58%. Among them, 161 contigs (99.94%, 189.02 Mb) were anchored into 16 pseudo-chromosomes (fig. 1*b*). The BUSCO completeness of the final *A. xuezhongi* assembly reached 98.70%, including 1,347 single-copy BUSCOs (98.50%), 3 duplicated BUSCOs (0.20%), 2 fragmented BUSCOs (0.10%), and 15 (1.20%) missing BUSCOs (table 1). We obtained mapping rates of 97.23%, and 97.61% for the Illumina and PacBio reads, respectively. Compared with three public Megachilidae genomes, the genome size of *A. xuezhongi* was smaller than that of *Megachile rotundata* (272.66 Mb) and *Osmia bicornis* (210.59 Mb), though the scaffold N50 length was much longer than for these prior assemblies (*Osmia bicornis*: 607.70 kb; *O. lignaria*: 5.48 Mb; *M. rotundata*: 1.70 Mb). These metrics emphasize the high quality of our assembly.

**Table 1:** Genome Assembly and Annotation Statistics of *Anthidium* x*uezhongi*

Elements	Current Version
Genome assembly	
Assembly size (Mb)	189.14
Number of scaffolds/contigs	23/168
Longest scaffold/contig (Mb)	17.44/12.52
N50 scaffold/contig length (Mb)	12.53/6.54
GC (%)	38.58
Gaps (%)	0.01
BUSCO completeness (%)	98.70
Gene annotation	
Protein-coding genes	10,820
Mean protein length (aa)	680.69
Mean gene length (bp)	6,974.90
Exons per gene	13
Exon (%)	26.74
Mean exon length (Mb)	358.90
Intron (%)	13.16
Mean intron length (Mb)	202.70

### Genome Annotation

RepeatMasker estimated that 13.74% (25.98 Mb) of the *A. xuezhongi* genome was masked as repetitive elements, including unclassified (7.37%), DNA transposons (4.33%), LTR elements (0.32%), LINEs (0.29%), and simple repeats (0.13%; supplementary table S3, Supplementary Material online). Retroelements and rolling-circles accounted for a small proportion of repetitive elements. The family TcMar-Tc1 (3.17%) was recovered as the most common DNA transposon element, making it a major component of the DNA transposons.

Altogether, we identified 385 ncRNAs, including 62 ribosomal RNAs, 59 miRNAs, 28 small nuclear RNAs, 4 ribozymes, 192 tRNAs, and 40 other ncRNAs (supplementary table S4, Supplementary Material online). The rRNA were classified into 47 ribosomal RNAs (5S and 5.8S) and 15 subunit ribosomal RNAs. The snRNAs were classified into 14 spliceosomal RNAs in six groups (U1, U2, U4, U5, U6, and U11), three minor spliceosomal RNAs in three groups (U4atac, U6atac, and U12), seven C/D box small nucleolar RNAs (snoRNAs), two H/ACA box snoRNAs, and one other snoRNA (supplementary table S4, Supplementary Material online).

The MAKER3 pipeline identified 10,820 protein-coding genes from 16,545 sequences in the *A. xuezhongi* genome with a mean length of 6,974.9 bp. The average number of exons, introns, and CDS per gene were 13.0, 11.3, 12.3, respectively, and their mean lengths were 245.3, 1827.5, 185.3 bp, respectively. The BUSCO completeness assessment for protein sequences reached 99.20% (*n* = 1,367), identifying 1,041 single-copy, 315 duplicated, three fragmented, and eight missing BUSCOs, indicating the high quality of these predictions. Diamond searches aligned 15,594 (94.74%) genes to the SwissProt and TrEMBL databases. Integrating with InterProScan and eggnog annotation results, 7,690 GO items, 6,730 KEGG pathway, 5,906 MetaCyc, 7,699 Reactome pathway, and 8,981 COG Functional Categories genes were assigned, respectively.

### Gene Family Evolution and Phylogenetic Relationships

Gene families were identified with OrthoFinder among 14 species, including 5 Apoidea species (see Materials and Methods). A total of 153,377 (94.60%) genes were assigned to 12,541 orthogroups (gene families). Among them, 1,897 orthogroups and 8,983 genes were assigned as being species specific; another 5,243 were orthogroups present in all species and 3,469 consisted of single-copy genes (fig. 1*c*). In *A. xuezhongi*, 10,091 (93.30%) genes were clustered into 9,251 gene families, of which 158 gene families were expanded, 482 gene families were contracted, and 31 gene families had experienced rapid evolution. Rapidly evolving gene families include several odorant receptors that may be linked to recognition of females during territorial behavior, but we could not meaningfully connect most to biological phenomena. With deeper sampling, we will better be able to isolate exactly which trends are specifically characteristic of both Anthidiini and this species. For these purposes, and to better interpret the ancestral state of the family Megachilidae, a genome of the early-diverging fideline bees will prove extremely valuable (Michener 2007).

Filtering with “symtest” resulted in 3,123 loci (1,826,179 amino acid sites), which were used to infer the phylogenetic tree in IQ-TREE, and with 100/100 support of all nodes. The phylogenetic reconstruction results are fully consistent with Peters et al. (2017) and Branstetter et al. (2017), supporting Formicidea as the sister clade of Apoidea. Parasitica, Vespoidea, Formicidea, and Apoidea were monophyletic groups, as expected. The Apoidea originated during the early Cretaceous period (131*–*138 Ma). Apidae and Megachilidae diverged during the late cretaceous (68.4*–*77.2 Ma). The common ancestor of the three Megachilidae taxa species arose in the Eocene period (44.5–47 Ma).

### Chromosomal Synteny

In comparing the chromosome-level genome assemblies of *A. xuezhongi*, *B. terrestris*, and *A. mellifera*, we captured 315 syntenic blocks (14,473 collinear genes). Strong collinearity in general among these three species revealed conserved chromosomal characteristics. However, a few chromosomes were derived from two or more chromosome segments, such as 16-chromosome from the fusion of segments of *B. terrestris* and *A. mellifera* chromosomes 2, 6, 7, and 11 (fig. 1*d*). However, large chromosomal differences have been found based on other behaviors, such as social parasitism within bumblebees (Sun et al. 2021), so we cannot rule out other factors at this time. More study with additional chromosomal assemblies will be necessary to test these possibilities.

## Materials and Methods

### Samples Collection and Sequencing

Adult specimens of *A. xuezhongi* were collected on June 19, 2020, at Chide Village, Pulan County (30.295_**°**_N 81.142_**°**_E; 3,934 m), Xizang/Tibet, China. The samples were deposited in liquid nitrogen and then stored at −80 _**°**_C before DNA was extracted. We removed the metasoma of all samples before sending them to the sequencing company (Berry Genomics, Beijing, China) to reduce potential gut microbe contamination. One individual specimen was used for each of PacBio, Illumina whole genome, Illumina transcriptome, and Hi-C sequencing.

Genomic DNA was extracted using Qiagen Blood and Cell Culture DNA Mini Kits, for PacBio and Illumina whole genome sequencing, respectively. PacBio sequencing was conducted on the PacBio Sequell II platform with an insert size of around 30 kb, using SMRTbell Template Prep Kit 1.0-SPv3. Sequencing libraries of 150-bp paired-end reads with an insert size of 350 bp were generated with a Truseq Nano DNA HT Sample preparation Kit (Illumina USA) on the Illumina NovaSeq 6000 platform. RNA sequencing was extracted by TRIzol Reagent, and the library was generated with TruSeq RNA v2 kit. Hi-C library was conducted on BGI MGISEQ-2000 platform with 150_**-**_bp paired-end reads.

### Genome Survey and Assembly

BBTools v38.29 (Bushnell 2014) was used for quality control on the raw Illumina data: duplicates removal with clumpity.sh; bbduk.sh to trim areas with quality scores lower than 20, reads shorter than 15 bp or more than 5 Ns, polymer trimming (>10 bp or poly-A/G/C tails), and overlapping paired reads. K-mer analysis and k-distribution were estimated by khist.sh (“21 k-mer”), and Genomescope v2.0 (Vurture et al. 2017) was then performed to estimate the size, heterozygosity, and content of repetitive elements of the *A. xuezhongi* genome with “-k 21 -p 2 -m 10,000.”

Flye v2.8.1 (Kolmogorov et al. 2019) was performed to assemble PacBio long reads with minimum overlap between reads of 1,000 bp and one round self-polishing. The Illumina sequences and PacBio assembly were aligned with Minimap2 v2.17 (Li 2018; Danecek et al. 2021). Removing heterozygous regions was performed by Purge_Dups v1.0.1 (Roach et al. 2018) with a cut-off at 60% identified contigs as haploids. Hi-C reads were subjected to quality control and aligned to the assembly by Juicer v.1.6.2 (Durand et al. 2016). Primary contigs were anchored into chromosomes using 3D-DNA (Dudchenko et al. 2017). Juicebox v.1.11.08 (Durand et al. 2016) was used to manually correct possible errors. BlastN-like MMseqs2 v11-e1a1c (Steinegger and S**ö**ding 2017) were performed to search potential contaminant sequences via the NCBI UniVec and nucleotide (nt) database.

Genome completeness was assessed using BUSCO v3.0.2 (Waterhouse et al. 2018), with the insecta_odb10 database (*n* = 1,367) as the reference gene set. In addition, to examine the utilization of clean data and completeness of assembly, we mapped clean reads of PacBio long reads and Illumina short reads to the final assembly with Minimap2, then calculated the mapping rate with SAMtools.

### Genome Annotation

Genome annotation mainly included repeat, ncRNA, protein-coding gene, and gene function annotations. RepeatModeler v2.0.1 (Flynn et al. 2020) was used to construct a de novo repeat library with the LTR discovery pipeline (-LTRStruct), which was based on a specific structure of repeats, and then, combined with Dfam v.3.3 (Storer et al. 2021) and the RepBase-20181026 (Bao et al. 2015) database to build the custom library. RepeatMasker v.4.0.9 (Smit et al. 2021) was applied to identify repeat regions against our custom library. Infernal v.1.1.3 (Nawrocki and Eddy 2013) and tRNAscan-SE v.2.0.8 (Chan and Lowe 2019) were applied to identify ncRNAs. MAKER v3.01.03 was used to predict protein-coding genes, integrating EVidenceModeler (EVM), based on three different strategies, including ab initio, transcriptome-based prediction, and homology-based prediction. For the ab initio gene prediction, we used BRAKER v2.1.5 (Hoff et al. 2016), which initially trained Augustus v3.3.3 (Stanke et al. 2004) and GeneMark-ES/ET/EP v4.48 3.60_lic (Brůna et al. 2020), simultaneously integrating evidence from transcriptome and protein homology to accurately model sequence properties. Hisat2 v2.2.0 (Kim et al. 2015) was applied to the transcriptome evidence in BAM alignments, and the relevant protein sources were obtained from OrthoDB v10.1 (Kriventseva et al. 2019) database. For the transcriptome-based prediction, the RNA-seq data were assembled with StringTie v2.1.4 (Kovaka et al. 2019) using the assembled genome as the reference. For homology-based prediction, we aligned the homologous proteins of six species, including *Solenopsis invicta*, *O. lignaria*, *Bombus vancouverensis*, *A. mellifera*, *Rhopalosiphum maidis*, and *Tribolium castaneum* (downloaded from NCBI database). The above results were integrated with Maker, with 1,7, 2 EVM weighting modules for ab initio, transcripts, and proteins, respectively.

We used two different approaches to annotate gene function: 1**)** Diamond v0.9.24 (Buchfink et al. 2015) applied to search the UniProtKB (Swissprot+TrEMBL) database with a sensitivity model (– more-sensitive -e 1e-6); InterProScan 5.41-78.0 (Finn et al. 2017) was performed to search Pfam (El-Gebali et al. 2019), Smart (Letunic and Bork 2018), Gene3D (Lewis et al. 2018), Superfamily (Wilson et al. 2009), and CDD (Marchler-Bauer et al. 2017) databases to predict protein domains; 2**)** based on Gene Ontology (GO) and Reactome pathways, eggNOG-mapper v2.0.1 (Huerta-Cepas et al. 2017) was used to search the eggNOG v5.0 (Huerta-Cepas et al. 2019) database. Finally, TBtools (Chen et al. 2020) was used to generate a visual diagram for genomic characteristics (LRT, LINE, SINE, DNA, GENE, GC, and Chr) of *A.xuezhongi* (fig. 1*a*).

### Gene Family Evolution

OrthoFinder v2.3.8 (Emms and Kelly 2019) was used to infer sequence orthology, based on high-quality protein annotation sequences downloaded from NCBI for 13 additional species: Ten Hymenoptera species (*Apis mellifera*, *Athalia rosae*, *Bombus vancouverensis*, *Chelonus insularis*, *Dufourea novaeangliae*, *M. rotundata*, *O. lignaria*, *Nasonia vitripennis*, *Solenopsis nvicta*, and *Vespa mandarinia*), one Diptera (*Drosophila melanogaster*), Hemiptera (*Rhopalosiphum maidis*), and a Coleoptera (*Tribolium castaneum*).

Protein sequences of single-copy orthologs were aligned with MAFFT v7.450 (Katoh and Standley 2013) using the L-INS-I strategy. To remove unreliable homologous regions, trimming was performed with BMGE v1.12 (Criscuolo and Gribaldo 2010) to alleviate compositional heterogeneity with the strict parameter “-m BLOSUM90 -h 0.4,” and then FASConCAT-g v1.04 (K**ü**ck and Longo 2014) was used to concatenate alignments. In addition, we used “–symtest-remove-bad –symtest-pval 0.10” command in IQ-TREE v2.0.7 (Minh et al. 2020) to determine and remove areas violating the assumptions of stationary, reversibility, and homogeneity (Naser-Khdour et al. 2019). We used LG “-m MFP –mset LG –msub nuclear –rclusterf 10” model in IQ-TREE, with 1,000 SH-aLRT (Guindon et al. 2010) and UFBoot2 (Hoang et al. 2018) replicates (-B 1000 –alrt 1000). Divergence times were estimated by MCMCTree package within PAML v4.9j (Yang 2007). Six fossils were downloaded from the PBDB database for node calibrations: root holometabolous: <382.7 Ma; the most common ancestor of Diptera and Coleoptera: <352.9 Ma; Hymenoptera: 205.6–212 Ma; Aculeata: 140.2–145 Ma; Apoidea: 130–125.5 Ma; Anthophila: 94.3–99.7 Ma. We used a JC69 substitution model, independent rates clock model, and approximate likelihood calculations. We repeated runs at least two times to make certain there was convergence (400,000 generations).

### Chromosome Synteny

MMsesqs2 v.11 was performed to identify interspecific chromosomal variation among *A. xuezhongi*, *Bombus terrestris*, and *A. mellifera* with an *e*-value threshold of 1e-10. The genome and annotation files of *Bombus terrestris*, and *A. mellifera* via NCBI. TBtools was used to generate a visual diagram for interspecific chromosomal (fig. 1*d*). At least eight genes were required to define a collinear block.

## Supplementary Material

Supplementary data are available at *Genome Biology and Evolution* online.

## Supplementary Material

evac014_Supplementary_Data
