# Supplementary material for: Chromosome-Level Genome Assembly of Anthidium xuezhongi Niu & Zhu, 2020 (Hymenoptera: Apoidea: Megachilidae: Anthidiini)
Source: Genome Biol Evol. 2022 Feb 12;14(2):evac014. doi: 10.1093/gbe/evac014 (PMC8850706; doi:10.1093/gbe/evac014)
Supplement: evac014_Supplementary_Data [file evac014_supplementary_data.zip › Supporting information.docx]

**Supporting information**

**Table S1.** Statistics of the sequencing data used for genome assembly.

**Table S2.** Genome assembly statistics for *Anthidium xuezhongi*.

**Table S3.** Repeat annotation in the *Anthidium xuezhongi* genome.

**Table S4.** Annotations of non-coding RNAs in the *Anthidium xuezhongi* genome.

**Table S5.** Potential contaminant results via the NCBI UniVec and nucleotide (nt) database.

**Fig. S1.** k-ker analysis of *Anthidium xuezhongi* basing k=21
