# Supplementary figures and images for: Chromosome-Level Genome Assembly of Anthidium xuezhongi Niu & Zhu, 2020 (Hymenoptera: Apoidea: Megachilidae: Anthidiini)
Source: Genome Biol Evol. 2022 Feb 12;14(2):evac014. doi: 10.1093/gbe/evac014 (PMC8850706; doi:10.1093/gbe/evac014)

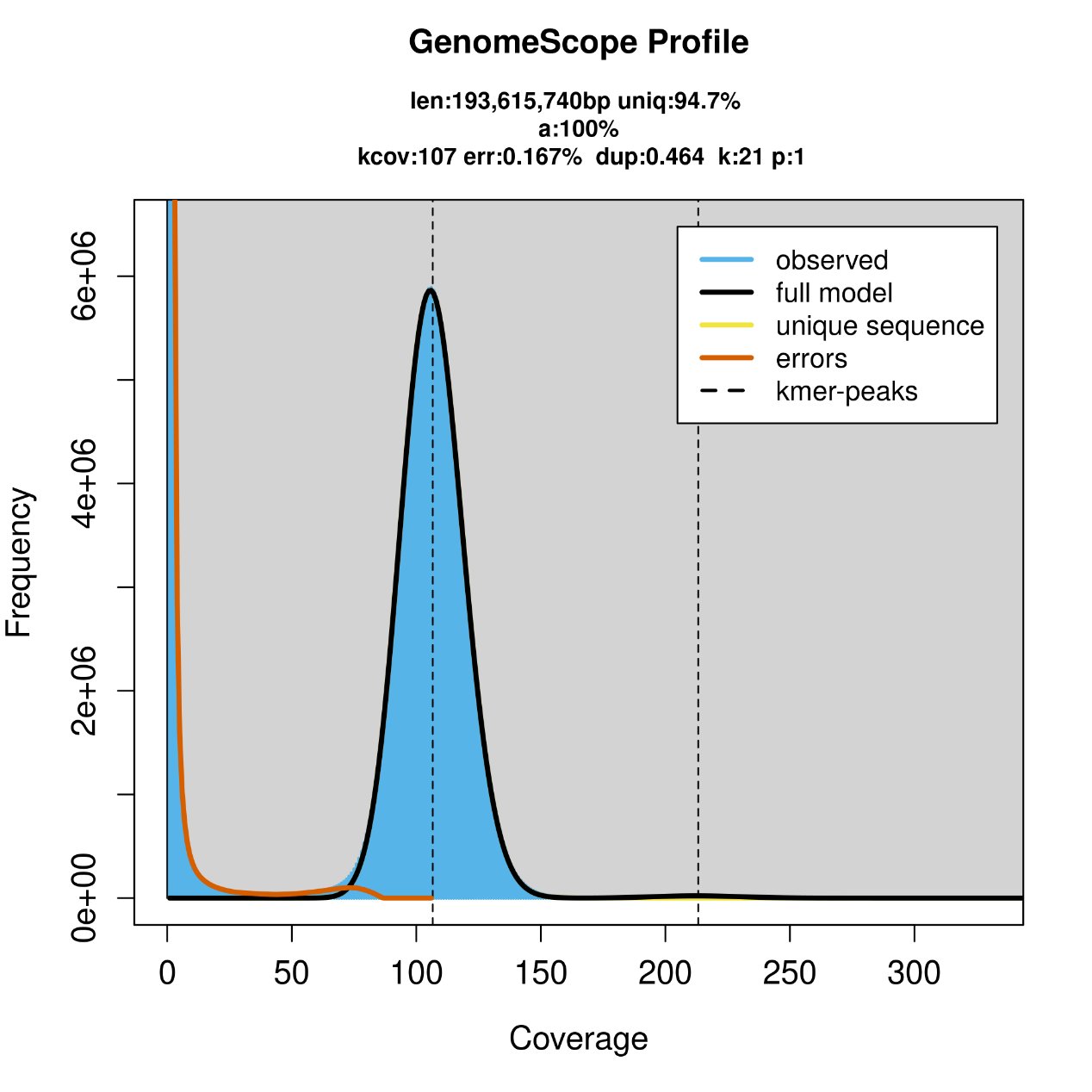


**Supplementary Figure 1.** K-mer analysis of *Anthidium xuezhongi* basing k=21

Supplement: evac014_Supplementary_Data [file evac014_supplementary_data.zip › supplementary_Fig.docx]
